# A pathway from fragmentation to interoperability through standards-based enterprise architecture to enhance patient safety

**DOI:** 10.1038/s41746-025-01442-3

**Published:** 2025-01-18

**Authors:** Zoie Shui-Yee Wong, Yang Gong, Shin Ushiro

**Affiliations:** 1 Graduate School of Public Health, St. Luke’s International University, Tokyo, Japan; 2 The Kirby Institute, University of New South Wales, Sydney, NSW Australia; 3 School of Medical Sciences, The University of Sydney, Sydney, NSW Australia; 4 School of Public Health, The University of Hong Kong, Hong Kong, China; 5 McWilliams School of Biomedical Informatics, the University of Texas Health Science Center at Houston, Houston, TX USA; 6 School of Medicine, Kyushu University, Fukuoka, Japan; 7 Japan Council for Quality Health Care (JQ), Fukuoka, Japan

**Keywords:** Health care, Information technology

## Abstract

Creating an ontology is the essential step in natural language processing (NLP). To improve patient safety in this era of generative AI, it is crucial to develop a standards-driven, ontology-based architecture for patient safety that can seamlessly integrate with health systems, thereby facilitating effective detection and monitoring potentially preventable harms in healthcare. This visionary, whole-system approach to patient safety addresses a significant gap in establishing resilient safety systems within the healthcare sector.

## Introduction

### The ongoing pursuit of patient safety

Envisaging “a world in which no one is harmed in health care, and every patient receives safe and respectful care, every time, everywhere,” the WHO *Global Patient Safety Action Plan 2021–2030* outlines a ten-year strategy to eliminate avoidable harm in healthcare. This strategy features global collaborative efforts and highlights the significance of harnessing digital technology and data usage^1^. The *Global Patient Safety Report 2024* also stresses the importance of harnessing information, research, and risk management^2^. Indeed, effective information management ensures a constant flow of information and knowledge to mitigate potential harm. However, such an endeavor faces significant practical challenges.

Incident reporting, a mainstay of capturing safety data in hospitals, is essential for implementing the Safety-I principle, which focuses on identifying and learning from errors. As of today, only one third of countries with incident reporting systems in their healthcare facilities actively report safety events to those systems. Moreover, challenges arise from the nature of voluntary reporting, underreporting, biased reporting, and the difficulties in analyzing large volumes of free-text data^3^. In contrast, the Safety-II paradigm changes the emphasis from reporting and learning about adverse events to highlighting what succeeds in healthcare, striving to gain insights from successes, even under challenging conditions. It encourages identifying and reinforcing actions that necessarily deviate from standard operating practices through resilience engineering^4^. Recognizing resilience and successful everyday practices, in turn, can facilitate better designed healthcare systems, improved communication, and the development of more robust processes.

A recent review suggests that by integrating Safety-I and Safety-II, an emerging Safety-III paradigm might offer great potential for improving diagnosis^5^. This integrated Safety-III approach emphasizes how human-system collaboration can maintain safety, including delegating decision-making to machines^5^. A scoping review has provided evidence on how AI, particularly natural language processing (NLP) systems, can be utilized across eight different harm domains, including diagnostic error, which is the most complex of the other patient safety harm domains^6^. It is defined by the Institute of Medicine as the failure to establish an accurate and timely explanation of a patient’s health problem or communicate that explanation to the patient^7^. Diagnostic safety^6^ hinges on three core concepts: (i) whether the healthcare systems can accurately identify a patient’s problem, (ii) whether a timely explanation is delivered, and (iii) whether that explanation is communicated effectively. The complexity of healthcare diagnostics - requiring timely coordination among various specialties, expertise, and operational stages - poses significant challenges to the agile deployment of knowledge gained necessary to achieve these goals. At present, though, there is emerging evidence of diagnostic improvement using novel AI^8,9^. For instance, ChatGPT models competed North American Spine Society clinical guidelines on cervical radiculopathy to provide safe and accurate recommendations^10^.

Despite all these advancements and new opportunities brought by large language models (LLMs), at present, most patient safety studies (regardless of whether they employ AI or not) have been siloed: their results are fragmented and unconnected, making it difficult to substantiate learning outcomes across different hospitals and countries. To achieve the goal of patient safety interoperability^11^ - reaching mutually agreed terminologies, ontologies, data, and analytics structures between different systems across different countries - one fundamental challenge lies in the need for a standards-based enterprise architecture (EA) design for patient safety.

Real-world data that is collected for patient safety comes in various formats and designs, which hinders interoperability. Currently, there is no commonly agreed-upon ontology or data structure for patient safety that can be applied across different settings. Consequently, useful applications of advanced AI and digital technologies, such as identifying diagnostic errors, providing real-time alerts, and assisting frontline staff to effectively communicate with patients, cannot be leveraged. The availability of “useful” databases that accurately capture errors and mitigate risks is essential to enhance hospital and diagnostic safety. This perspective paper discusses the evidence around state-of-the-art patient safety science and digital transformation. It is timely and novel, aiming to provide a forward-looking perspective on the importance of EA in safety-oriented organizations and to stimulate future approaches to adopting EA. We stress the necessity for research to concentrate on a networked knowledge ecosystem, underscoring the significance of connecting interrelated concepts in patient safety through an ontological method. Consequently, this allows NLP and AI to enhance interoperable patient safety. By providing a summary of recent advances in NLP models to patient safety reporting, this perspective proposes a visionary, whole system approach to enhancing patient safety via integration of key safety principles.

### Recent advances in LLM for incident reports/events learning

The *Global Patient Safety Report 2024* indicated that 32% of countries and 60% of healthcare facilities participate in patient safety incident reporting and learning systems^2^. A content analysis study investigating approximately 2900 incident reports in the US found major reporting inconsistencies^12^. Although the 2015 Minimal Information Model for Patient Safety (MIM PS)^13^ provides a global reference for reporting data in both structured and unstructured formats, these are only guideline recommendations, and significant variations are still found from one institution to another, particularly in narrative notes. At present, open access datasets are available from the U.S. FDA’s Adverse Event Reporting System (FAERS) and Manufacturer and User Facility Device Experience (MAUDE), the Vaccine Adverse Event Report System (VAERS)^14^, the national Japan Council for Quality Healthcare (JQ)’s “Project to Collect Medical Near-Miss and Adverse Event Information”^15^ and the “Project to Collect and Analyze Pharmaceutical Near-Miss Events”^16^. These valuable public resources enable informatics research in incident report areas.

Over the past decade, patient safety informatics research has increasingly employed NLP to analyze incident reports in various ways, for example unsupervised learning^17^, similarity search^18,19^, and text classification by machine learning^20–22^ and neural networks^23^. Wong et al. developed annotation methods for incident reports that teach NLP models to recognize medication-error sensitive named entities and attribute extraction^24^. A series of patient safety studies has revealed the learning value of exploring publicly available patient safety datasets^25–27^. NLP models scaled for distinguishing between intended and actual acts detect the discrepancies between the intended delivery and what is actually delivered to the operation^28^, which allows transformer-based LLMs to automatically distinguish error from non-error drug concepts. These efforts have enabled the creation of gold standard, manually annotated incident reports, and also provide the world’s most extensive, open-resources corpus of machine-annotated incident reports on medication errors^29^. A proof-of-concept digital tool was also released to the public to demonstrate how to automatically structure medication-related incident reports with an LLM^30^. Recent MAUDE studies also show potential in using generative AI to improve data extraction, transformation, and cause-effect visualization stored in a vector database, facilitating event retrieval and comparison based on a similarity search^31–33^.

### Ontological approach to building data structures for patient safety

A common international understanding of terms and concepts in regard to patient safety is presently lacking^34^. While the WHO’s International Classification for Patient Safety (ICPS version 1.1) serves as a conceptual framework for patient safety, it merely references essential patient safety concepts and does not function as an ontology. Ontology is a detailed map that categorizes a networked of concepts in a particular area of knowledge. Its purpose is to define the relationships between different concepts (e.g., drug, patient characteristic, allergy level), showing how they are connected to each other. Validated guides^35^ and open-source ontology frameworks, such as Portege^36^, are resources for knowledge-based systems.

Applied to biomedical domains, ontology has successfully structured complex medical and biomedical data. Significant translational advancements in biomedical domains have been able to effectively capture the growing volume and complexity of data. Unfortunately, ontologies focused on patient safety have not received the same level of attention as bioinformatics domains, such as those relating to living organisms, including plants and animals.

The challenge lies in the fact that most patient safety events involve complex, interconnected concepts, resulting in much of the context being captured as unstructured narrative safety notes. This makes it difficult to apply an ontological approach, i.e. the essential first step of NLP, which is to organize the “*things of interest*^24^” within the complexity of incidents. Although there were early attempts to map knowledge representation in patient safety^37,38^, these efforts were not sustainable. A systematic review published in 2022 reported patient safety classifications and ontologies^39^; only two general domain ontologies were available in the literature, namely Ontology of Adverse Events (OAE)^40^ and Patient Safety CAtegorical Structure (PS-CAST)^41^. Other ontologies covered areas of medication, pharmacovigilance, cardiology, clinical communication, vaccine, neuropathy, and kidney disease^39^. These are all specifically focused and operate in silos. These findings reflect a lack of coordinated effort towards developing an ontology for safety and risk management. Indeed, a well-structured ontology can facilitate reporting, analysis, and learning while enabling reasoning and root cause analysis. We believe that all existing ontologies, as well as any future ones, should ideally be consolidated into a comprehensive ontology to promote universal knowledge. Examples such as the person-centric ontology (HSPO) are designed to represent and integrate information about an individual across multiple domains^42^. Such an approach is transferable to a patient-centered ontology design for a patient safety knowledge graph. Furthermore, when designing the data element, a “fit for purpose” principle that considers data quality should be utilized to ensure that data items sufficiently support their intended use^43^.

Recently, common data models (CDMs), such as the Observational Medical Outcomes Partnership (OMOP) and Fast Healthcare Interoperability Resources (FHIR), have demonstrated interoperable successes worldwide through the standardization of the logical infrastructure of health IT systems. These CDMs have provided new insights into how complex free text and structured data within hospitals should be registered, managed, and reused across different healthcare contexts. OMOP includes data elements useful in assessing patient safety, such as adverse event detection, comparative safety studies, post-market surveillance, and patient outcomes. However, OMOP is not designed to improve patient safety. Despite some pilot studies exploring necessary data element models in event reports^44^, no common data structure yet exists for the comprehensive registration of patient safety events in hospitals that consider the Safety I and Safety II paradigms.

Currently no universally accepted ontology, data structure or toolkit that can organize potential high-risk events for later preemptive analysis. This highlights the need to develop an inclusive framework tailored for patient safety detection and management, as well as the need to establish an open, collaborative platform where contributors can enhance and expand the framework - similar to the approach adopted by open-source health software platforms. Existing common terminologies/classifications/ontologies in patient safety can be used as the foundational building blocks, such as the Joint Commission on Accreditation of Healthcare Organizations (JCAHO)’s “Patient Safety Event Taxonomy” and “Medical Dictionary for Regulatory Activities” (MedDRA).

### An architecture for safety-oriented healthcare organizations

**Fig. 1:**
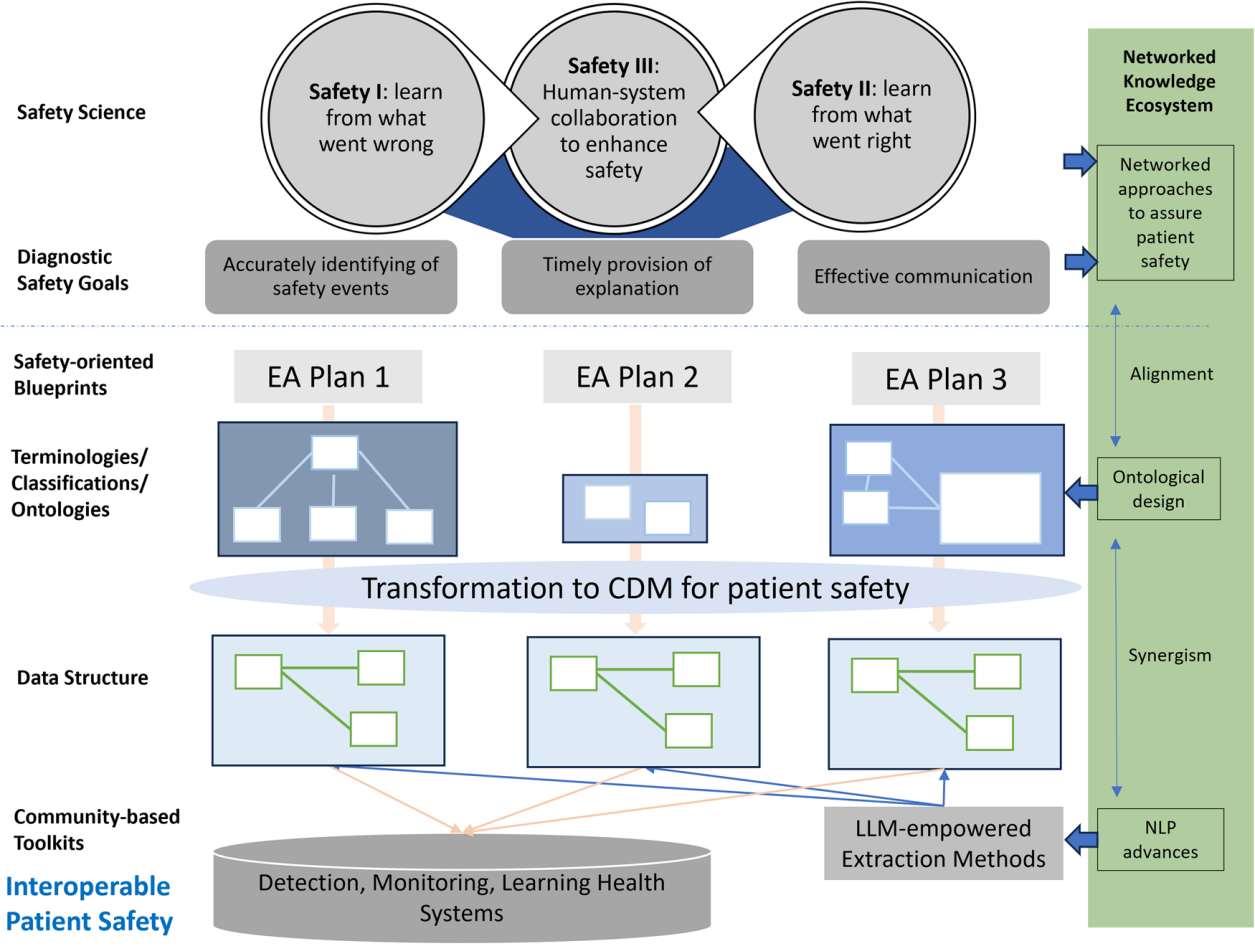
Interoperable patient safety blueprint. Achieving interoperable patient safety across health systems begins with awareness of safety science principles and an understanding of diagnostic safety goals. Safety-oriented organizations, depending on their business scope, should design a suitable Enterprise Architecture (EA) plan that incorporates the necessary data, information, and objects into their blueprint. This blueprint should adopt appropriate standards that align with a common data structure for patient safety. This enables subsequent community-based approaches, including automated large language model (LLM) extraction methods to retrieve standards-based content from clinical notes, which in turn facilitate interoperable patient safety through detection, monitoring, and the empowerment of learning health systems. On the right-hand side, this figure illustrates a research shift towards a networked knowledge ecosystem, outlining how multilayered and interconnected patient safety elements should be structurally represented through ontological design. Safety data is then processed using advanced NLP and AI, which convert it into a commonly agreed-upon structure, facilitating interoperable patient safety across safety-oriented systems. EA - enterprise architecture; CDM – common data model; LLM – large language model.

We advocate that an EA approach should be considered when designing a blueprint for patient safety. The International Telecommunication Union (ITU) has emphasized EA’s importance in digital transformation^45^. EA is also a critical component of governmental digital health design^46^ and digital health maturity^47^. The EA process is often ignored until a new value proposition comes to the surface. Figure 1 presents the patient safety blueprint design for safety-oriented healthcare organizations.

In the field of information technology, architecture is defined as a structured vision that outlines how systems and components are organized and interact effectively. Architecture provides an integrated view of the system being investigated, and architecture at the level of an entire organization is referred to as enterprise architecture^48^. “Enterprise” here refers to a healthcare provider that aims to provide quality and safe healthcare to patients. The concept of enterprise can be extended to include partners, suppliers, customers, and internal business units. An EA for patient safety encompasses a coherent set of principles, methods, and models used to design and implement the aspects of an organization’s structure, business processes, and information systems. There are open resources available for EA, for instance, step-by-step guidelines for EA design are available in the open domain and published by WHO/ITC^49^. Furthermore, validated EA methods, such as the Open Group Architecture Framework (TOGAF) and the Zachman Framework, are useful for guiding enterprise architects in designing blueprint plans, could potentially enhance patient safety.

The Zachman Framework was developed from extensive experience in managing change within complex systems, namely the aviation industry. Consequently, it has the potential to serve as an appropriate framework for safety systems in the healthcare sector. In this paper, we explain our vision to develop a blueprint for patient safety architecture utilizing the Zachman Framework, which comprises, a two-dimensional, 6 × 6 classification system of 36 cells that each describe one perspectives of an enterprise. These perspectives encompass six stakeholder viewpoints (“planner”, “owner”, “designer”, “builder”, “subcontractor”, and “enterprise”) and six interrogatives (“what”, “where”, “who”, “when”, “why” and “how ”). We briefly outline how these interrogatives apply to safety-oriented organizations in Table 1.

**Table 1:** A concise explanation to develop a blueprint for patient safety architecture utilizing the Zachman Framework

Illustrative Applications in Safety-Focused Health Organizations
The “**what**” aspect consists of the data, information, and objects that are involved in the business process. Enterprise architects at safety-oriented health organizations should determine which data items are relevant based on careful consideration of business operation. The data may encompass patient demographics, medication logs, routine operation data (that reveals how care teams adapt to challenges and drive positive outcomes), incident specifics (frequently documented in incident reports), and timestamps of when incidents occur. Incidents within hospitals frequently encompass medication errors, falls, or complications relating to blood products, the designer would need to consider the characteristics of these incidents to incorporate the necessary data items. Regarding the scope of data, incidents associated with community pharmacies, including medication errors linked to prescriptions dispensed by these pharmacies, should be integrated into the data analysis, provided that they are pertinent to the business model. Similarly, adverse events related to medical devices may or may not be considered part of the plan depending on business function. The specific data items may vary based on the enterprise’s health services and clinical processes. Pertinent business information might include the findings of incident root cause analysis, trend analyses (e.g., dashboards displaying incident types and frequencies), or process-specific risk profiles (e.g., high-risk scenarios of older patients with peripheral neuropathy were more likely to be associated with fall incidents^62^). Business objects include entities such as quality improvement (QI) centers, patient safety professionals, safety alerts (which could be integrated into decision support systems), and hospital safety protocols.
The “**where**” aspect considers the physical and virtual networks of care delivery. Hospital care is a complex process involving interconnected clinical specialties. Depending on the service design, the system may include external pharmacies or in-house pharmacies as part of the overall entity. These elements significantly influence the achievement of business functions and goals.
The “**who**” aspect emphasizes the individuals driving healthcare operations, including healthcare professionals, assistants, and clinical specialists, as well as their hierarchical structure of teams and units.
The “**when**” aspect addresses the timing and frequency of business processes, including healthcare operating schedules and workflows.
The “**why**” aspect considers the rationale behind the introduction of digital services and platforms that are relevant to the business objectives of safety-oriented organizations.
The “**how**” aspect examines the mechanisms through which the healthcare business operates, and the clinical processes involved in delivering services.

Ultimately, an EA plan provides a formal, holistic description of the entire enterprise as an interconnected system of systems. It details the components and their integration, illustrating how people, processes, technology, and data interact and collaborate to achieve safety-oriented healthcare objectives. Enterprises that successfully integrate EA into their digital health services would often have fully trained enterprise architects or an EA taskforce to ensure proper structuring of components, effective management of their interrelationships and monitoring their evolution over time.

By connecting standardized data structures, terminologies, and tools, knowledge sharing is promoted within a community of experts operating within the same technical ecosystem. When data becomes interoperable, seamless data exchange between applications is possible. These standards-based capabilities support the organization and individuals in optimizing knowledge exchange and advanced analytics central to optimization and innovation. For effective patient safety, EA planning should adopt inter-sectoral and sociotechnical approaches to translate both clinical and patient needs into specific functional and technical requirements. These requirements guide the collaborative development and deployment of digital health services. Additionally, involving patients in safety detection and feedback is crucial.

A comprehensive patient safety ontology and common data models for recording patient safety events in hospitals remain undeveloped, highlighting significant research gaps. The advantage of the ontological approach for patient safety is that it provides a representation of semantic information and enables a wealth of information to be captured through reporting^38^. With recent advances in LLMs, automatic extraction of narrative notes mapped into a knowledge graph network has become possible. We call for advancing an ontological approach to a whole-system, strategic infrastructural blueprint design for hospital patient safety, considering both Safety-I and Safety-II operations to empower the Safety-III paradigm.

A patient safety learning system refers to a healthcare system where knowledge generation processes can be seamlessly embedded into daily practice to facilitate continual improvement of care processes^50,51^. Having a whole-system, standards-based design as a foundation, a patient safety learning system can harness the knowledge gained from narrative incident reports, enabling multifaceted, AI-supported incident analysis, case monitoring, and frontline education to yield better patient outcomes. This will also enable direct performance outcomes in root-cause analysis, clinical users’ learning outcomes, and hospital incidence reduction^52^.

In healthcare, an ontology that defines medical terms, procedures, and drug names can enhance an NLP system’s ability to interpret clinical documents or medical records. By providing a structured framework for understanding domain-specific language, LLM-empowered toolkits can be designed to extract essential content, based on the ontology to improve information extraction, such as entity recognition and relation extraction. Furthermore, recent advances in generative AI enables semantic understanding and text regeneration according to standards.

Considering the successes of engaging global communities to open CDM, such as OMOP^52^, and FHIR^53^, this standards-based patient safety common data structure should bring together global efforts to be tested and validated in real-world cases, enabling the detection of hospital incidents and the identification of successful practices. With recent advances in LLMs creating additional technical possibilities, it is imperative for the patient safety community to have mutual agreement upon the design of the standards-based common data model to ensure maximize potential in coordinated patient safety improvement. Defining NLP problems and creating evaluation methods in the patient safety context, which inherently emphasizes root-cause analysis and requires advanced reasoning and investigative skills remains challenging, even with generative AI. However, the growing volume of safety data, e.g. incident reporting and learning resources, provide an opportunity to scale clinical free text into structured, ontological formats that can support patient safety initiatives. This approach will provide a reference patient safety data structure, enabling other patient safety researchers to contribute to the completion of the patient safety ontology. A CDM approach like this is designed to be globally interoperable to maximize the international impact on patient safety.

### Future research and challenges

This paper presents a progressive blueprint for a patient safety architecture designed to achieve Safety-III, which aims to transform the registration and management of adverse events (Safety-I) and successful outcomes (Safety-II) within the healthcare sector. It begins with a review of recent advances in LLMs applied to incident report information extraction, demonstrating how these cases can transform patient safety. Our vision is to create a standard that employs a patient-centered framework to systematically register incidents through ontological approaches, which subsequently allows the community to enrich the data structure by contributing to enabling toolkits. This also supports a learning health organization where digitally competent health professionals deliver digitally enhanced care in partnership with digitally literate patients and consumers. To do so, an organization-wide patient safety approach must be integrated to enable a standards-based ICT infrastructure with detection, monitoring and analysis capabilities. We envision that digital tools and data for patient safety will mature and become interoperable through a platform with standards for interoperability and quality of all data, tools, and services that are published and implemented.

Thematic analysis identified four main drivers for increasing the interoperability of electronic systems in health^54^, which includes improving patient safety. Despite the importance of EA in the role of digital transformation^45^, studies have shown that implementing EA can be challenging^46,55^. Without EA, many systems have become overly complex and misaligned with business needs. Drawing from insights gained during past EA implementations, we recognize the necessity to enhance high-level decision makers’ understanding of EA and to emphasize the importance of communication and leadership skills among enterprise architects. Barriers associated with the interoperability of processes are likely to come from system capability, hospital policy, and staff culture^54^. Through extensive interviews with architects and managers, the study revealed that a smoothly operated EA initiative would include incentivizing clinicians to acquire pre-designed systems (based on common requirements), a fit-for-purpose approach to cater to both standardization and local needs and strong patient engagement throughout the process^56^. Another area requiring further investigation is the ethical use of LLM in patient safety, focusing on transparent disclosure to ensure accountability and trust. While this paper does not center on data governance and regulation, we call on patient safety authorities to thoroughly consider regulatory facets, including federated learning^57^, cross-border data policies^58,59^, protecting patient-sensitive information^60^ and the oversight roles of authorities, hospitals, and health information ethics. Furthermore, investigating offline, non-cloud LLMs could help prevent leaks of sensitive patient data. LLMs may introduce hallucinations and biases^61^. Exploring bias detection, mitigation and reduction techniques, such as error analysis, fairness-aware learning and retrieval-augmented generation, are also crucial research focuses.

In this era of generative AI, while many are striving for quick success, we instead advocate for an enterprise-based blueprint to integrate disconnected approaches. Transforming fragmentation into interoperability requires raising standards-based EA awareness, implementing shared data models, and fostering open community initiatives. This will enable the synthesis of the impact of patient safety in healthcare and help achieve the ultimate goal of preventing avoidable harm. With an ontological approach to patient safety, we foresee that guidelines, annotated datasets, models, and tools will serve as invaluable assets to explore more challenging topics in the digital transformation of a wide range of hospital incidents. Substantive efforts to understand hospital safety, from what went wrong to what went right, as well as accurate AI modeling and seamless human-computer interaction, are essential. This direction is significant because it will unify global norms, consensus, and standards for key concepts, preferred term definitions, measurement, and reporting of near misses and adverse events in healthcare, allowing for international benchmarking.

## Data Availability

No datasets were generated or analysed during the current study.
